# Supplementary material for: Barley SIX-ROWED SPIKE3 encodes a putative Jumonji C-type H3K9me2/me3 demethylase that represses lateral spikelet fertility
Source: Nat Commun. 2017 Oct 16;8:936. doi: 10.1038/s41467-017-00940-7 (PMC5643332; doi:10.1038/s41467-017-00940-7)
Supplement: Supplementary file 1 — Supplementary Information [file 41467_2017_940_MOESM1_ESM.pdf]

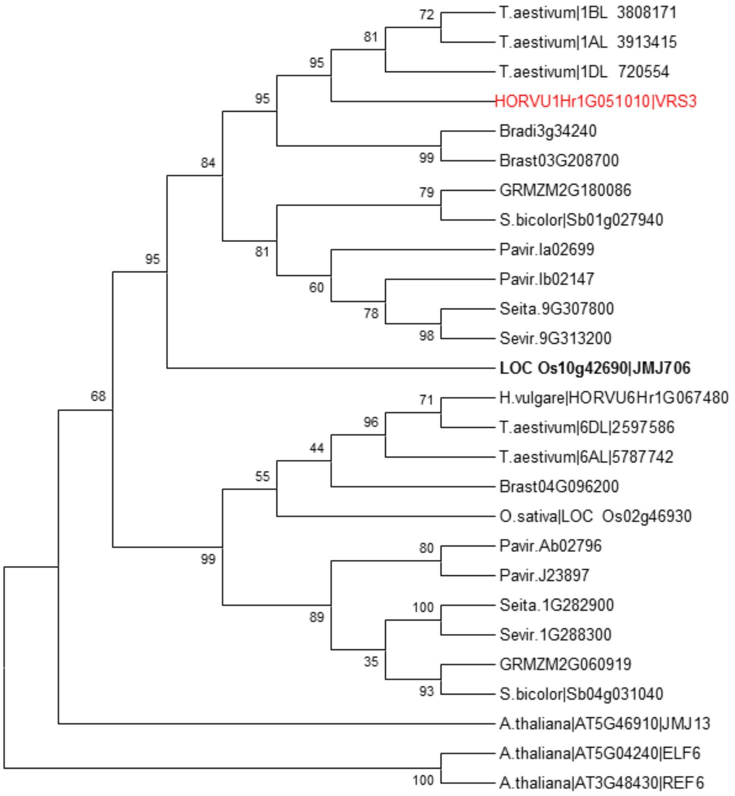

Supplementary Figure 1: *VRS3* Phylogeny across the Triticeae. Numbers show the percentage bootstrap positioning of the clade.

a

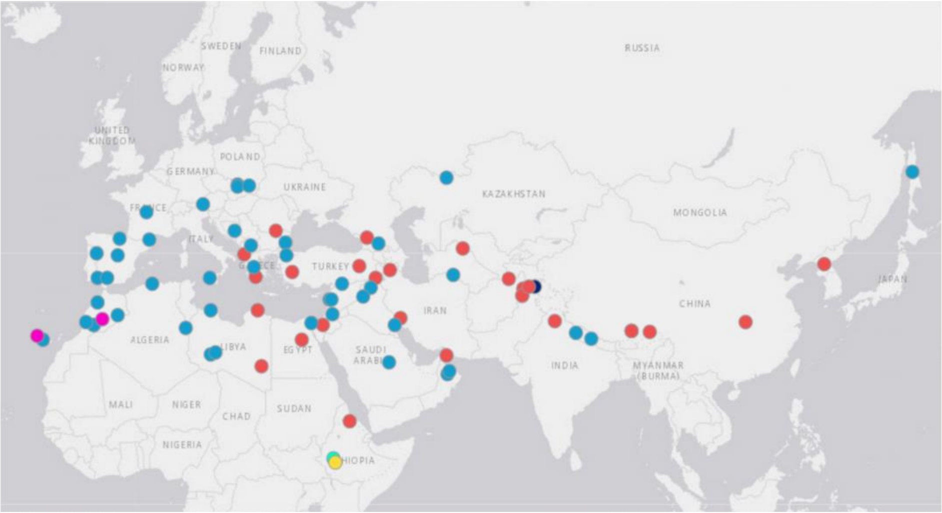

b

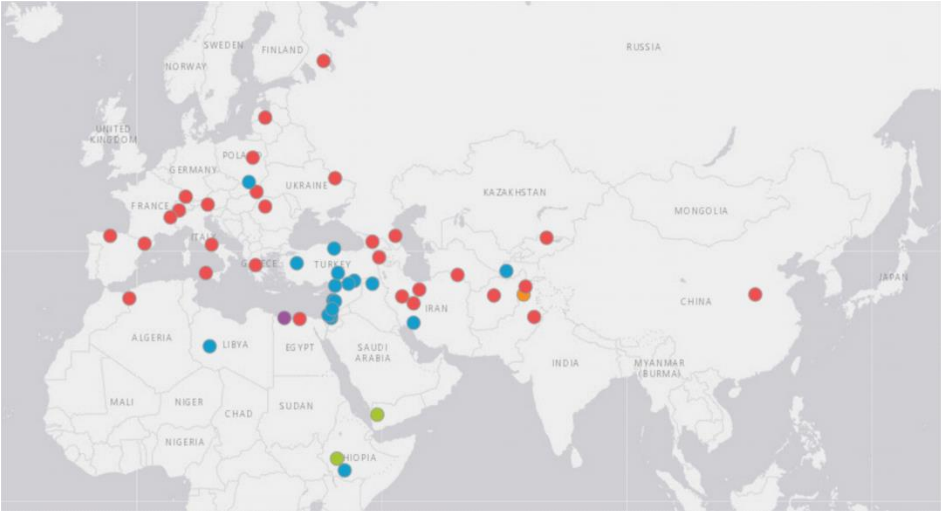

Supplementary Figure 2: Distribution of domesticated *VRS3* haplotypes in geographical space. (**a**) Six-rowed landraces (Red: haplotype 45; Blue: haplotype 46 from Main Text Figure 3; Pink: haplotype 1; Dark Blue: haplotype 18; Turquoise: haplotype 19; Yellow: haplotype 20). (**b**) Two-rowed landraces **(**Red: haplotype 45; Blue: haplotype 46; Green: haplotype 14; Purple: haplotype 11; Orange: haplotype 17). Accessions including geolocations are listed in Supplementary Data 5 and haplotypes are described in Supplementary Data 6. The maps were generated at ArcGIS (http://doc.arcgis.com/en/arcgis-online/reference/static-maps.htm)

This image is used with permission from Environmental Systems Research Institute, Inc. (Esri). Copyright © 2017 Esri, ArcGIS Online, Creative Commons, and the GIS User Community. All rights reserved.

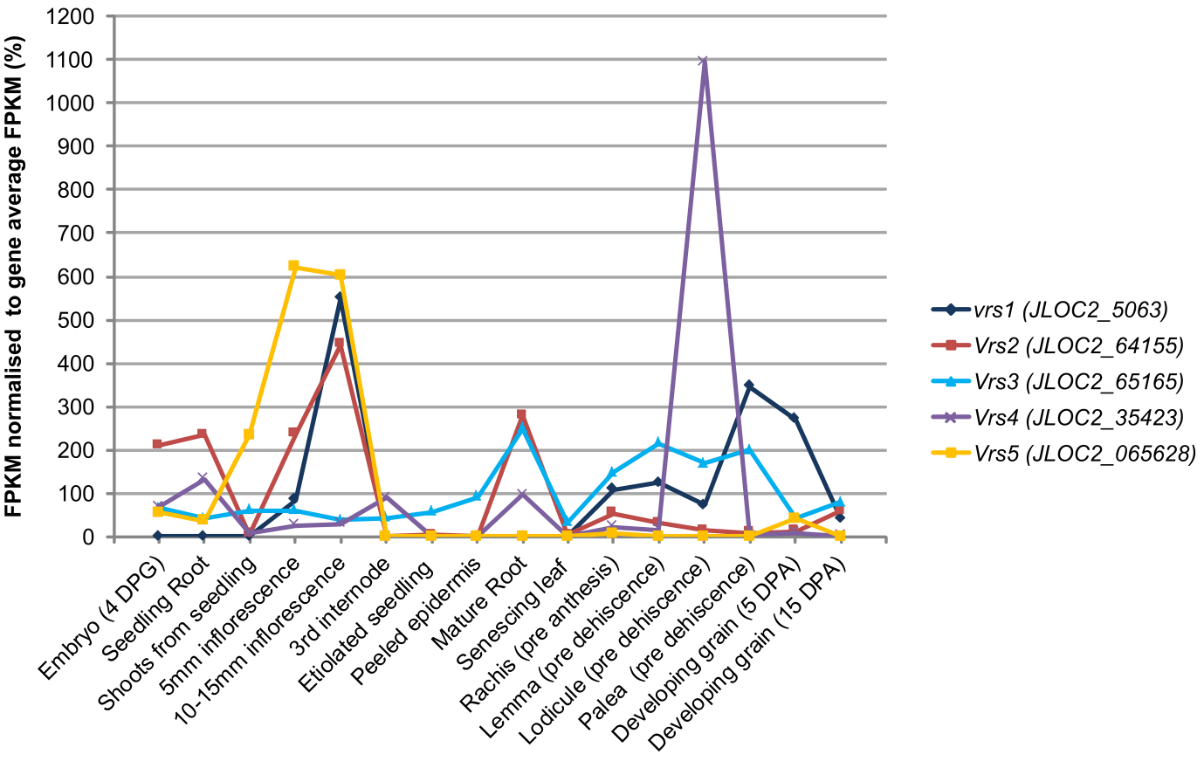

Supplementary Figure 3: Barley atlas gene expression. Tissue expression profile of *Vrs3.w* in comparison to row-type loci *vrs1, Vrs2*, *Vrs4* and *Vrs5* (*Int-c*) in the six-rowed cultivar Morex. Tissues are ordered according to developmental time of the plant at sampling. Values are relative to the average expression of the gene across all 16 developmental tissues. DPG, Days post-germination; DPA, Days post anthesis.

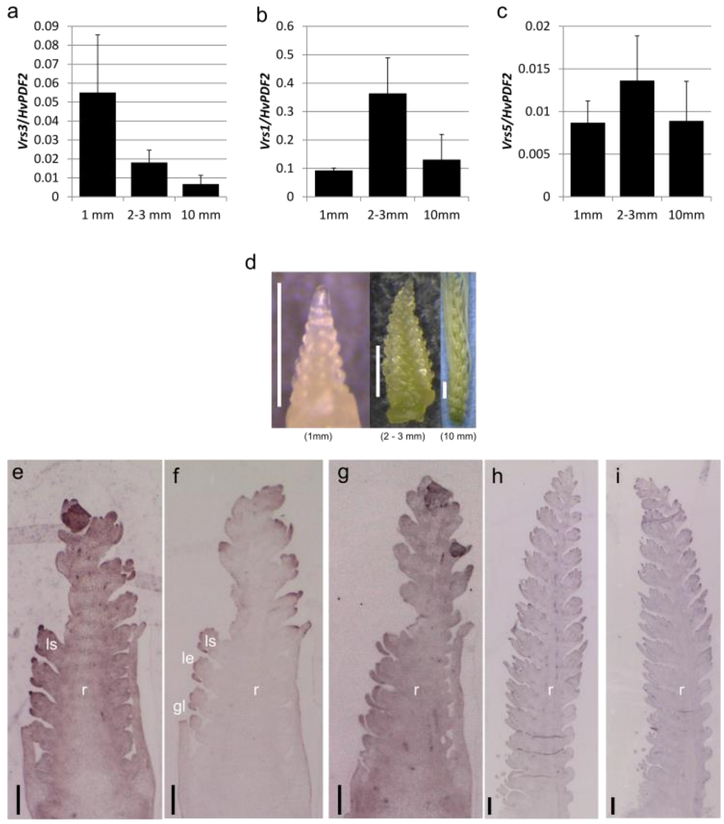

Supplementary Figure 4: *VRS3* gene expression. (**a-c**) Wild-type expression of *Vrs3*.w, *Vrs1*.b and *Vrs5*(*int-c*.b) relative to the reference gene *HvPDF2* across the three stages of developing Bowman inflorescences, triple-mound (TM; spike length 1mm), lemma primordium (LP; spike length 2-3mm), and white anther (WA; spike length 10mm). Mean normalized expression ± S.E across 3 biological replicates is shown. (**d**) Representative photos of TM, LP and WA stages. Scale bars, 1mm. (**e-i**) RNA *in situ* hybridization analysis of *VRS3* in Bowman spikes. (**e,f**) Longitudinal sections at lemma primordium hybridised with (**e,f**) antisense VRS3 probe showing signal in the lateral glume and lemma primodium in serial sections and (**g**) sense probe. ls, lateral spikelet; le, lemma; gl, glume. Scale bars, 100 µm (**h,i**) Longitudinal sections of awn primordium stage hybridised with (**h**) antisense VRS3 probe and (**i**) sense probe. Scale bars, 200 µm.

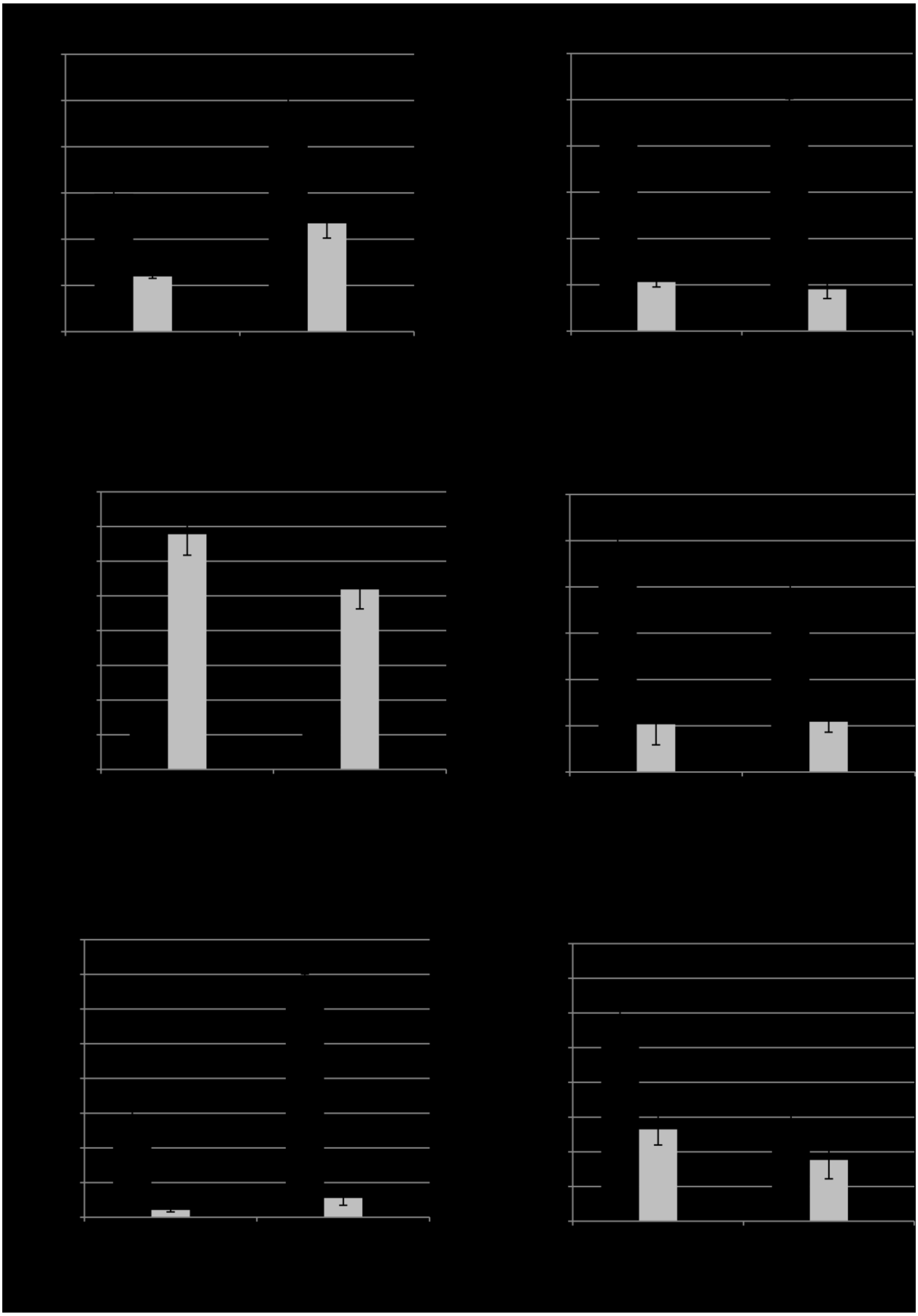

Supplementary Figure 5: qRT-PCR validation of differentially expressed genes identified in the RNA-seq experiment. *vrs3* mutant (BW902 (*vrs3.*f) shown in grey in comparison to Bowman (WT) in black

at the two developmental stages assayed, AP (awn primordium, 5mm-spike) and WA, (white anther, 10mm-spike). **(a-f)** qRT-PCR validation of **(a)** *HOX-2*, (b) *TCP FAMILY TRANSCRIPTION FACTOR*, (c) *LONELY GUY-LIKE*, **(d)** *CYTOKININ OXIDASE/DEHYDROGENASE*, **(e)** *TERMINAL FLOWER 1-like* and **(f)** *TREHALOSE 6 PHOSPHATE PHOSPHATASE*. *HvActin* was used for normalization. X-axis shows the inflorescence developmental stages in which the qRT-PCR was carried out. Y-axis shows the relative expression level based on ΔCt (cycle threshold) calculation. Mean ± S.E of three biological replicates is shown. Significant differences among the samples were established using Student's t-test (two-tailed).

Glasshouse

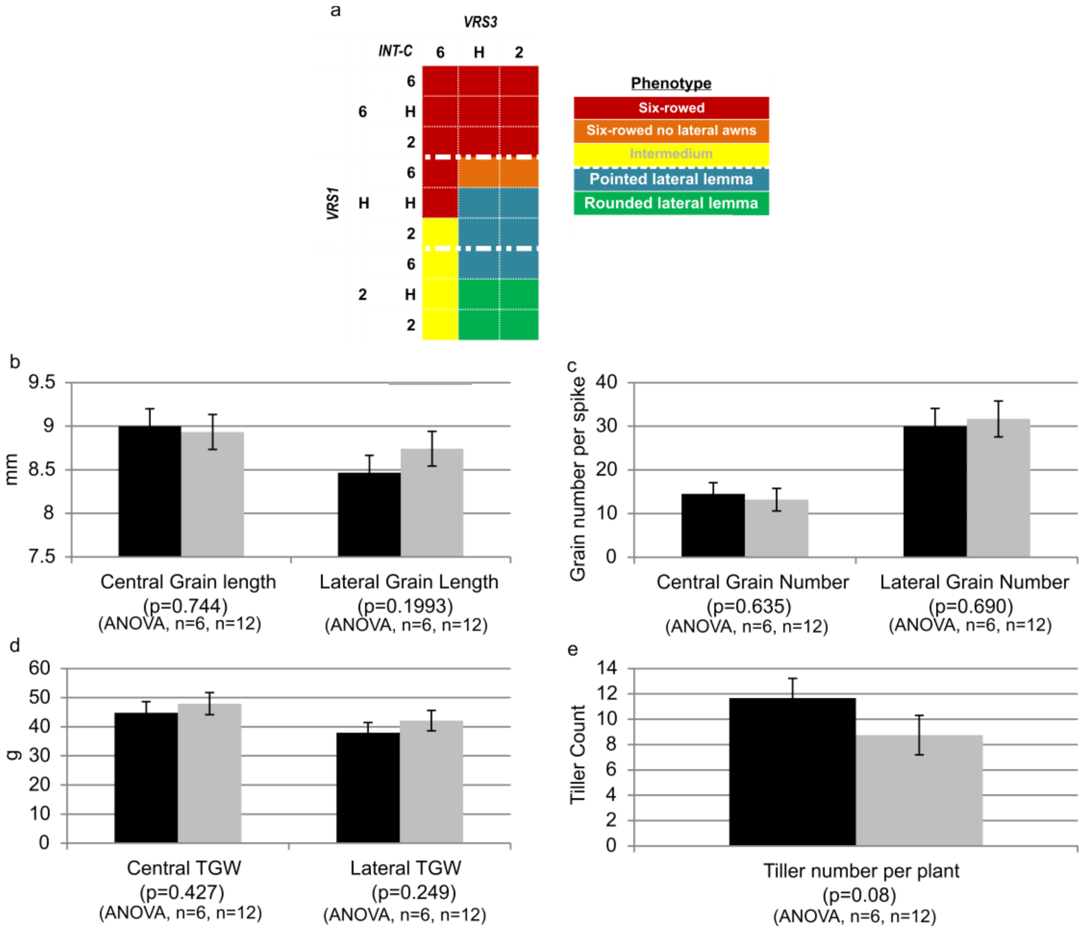

a
VRS3
INT-C
6 H 2
VRS1
6
H
2
Phenotype
Six-rowed
Six-rowed no lateral awns
Intermedium
Pointed lateral lemma
Rounded lateral lemma
b
mm
Central Grain length
(p=0.744)
(ANOVA, n=6, n=12)
Lateral Grain Length
(p=0.1993)
(ANOVA, n=6, n=12)
c
Grain number per spike
Central Grain Number
(p=0.635)
(ANOVA, n=6, n=12)
Lateral Grain Number
(p=0.690)
(ANOVA, n=6, n=12)
d
g
Central TGW
(p=0.427)
(ANOVA, n=6, n=12)
Lateral TGW
(p=0.249)
(ANOVA, n=6, n=12)
e
Tiller Count
Tiller number per plant
(p=0.08)
(ANOVA, n=6, n=12)

Field

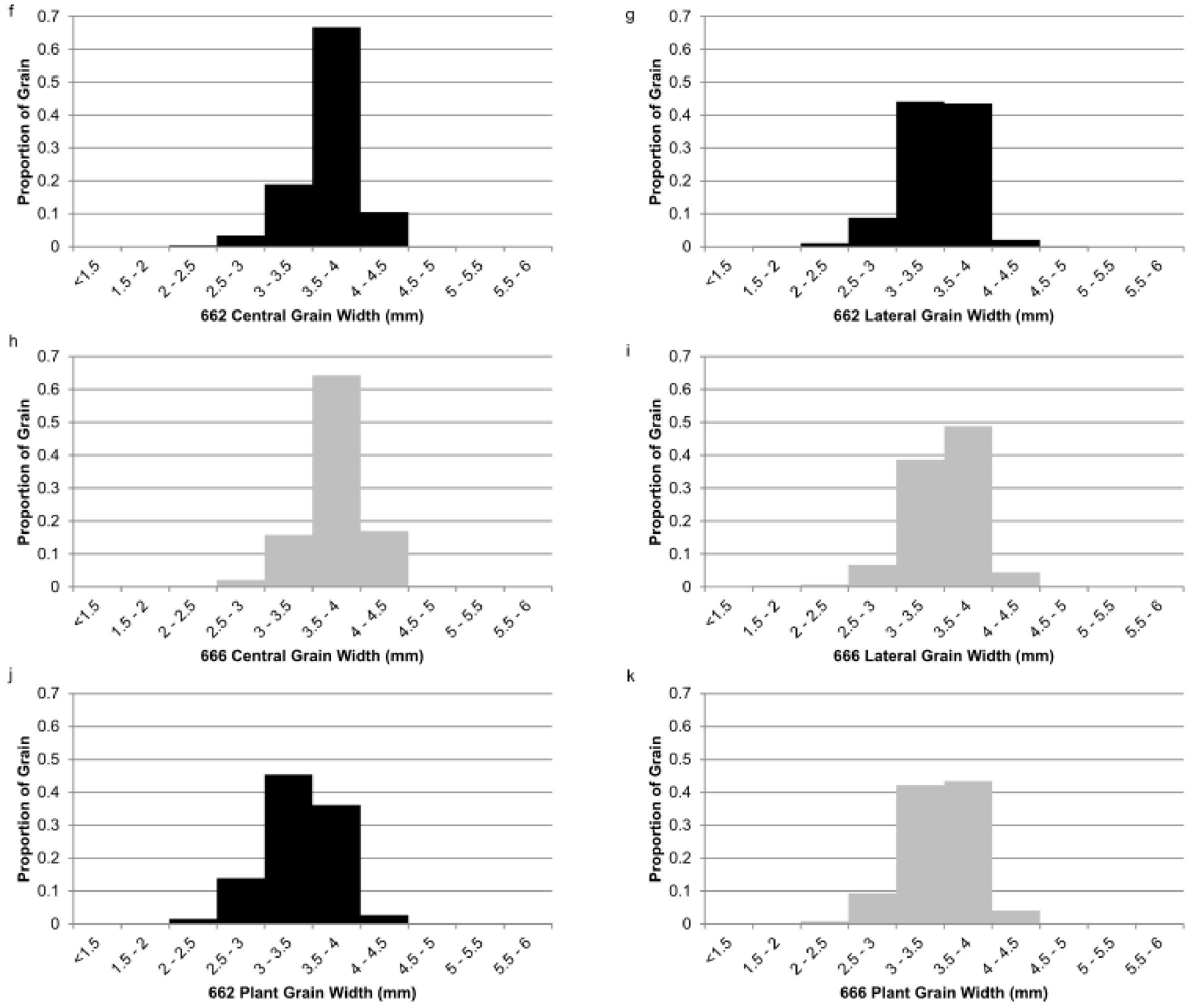
f
g
h
i
j
k
Proportion of Grain
0 0.1 0.2 0.3 0.4 0.5 0.6 0.7
<1.5 1.5 - 2 2 - 2.5 2.5 - 3 3 - 3.5 3.5 - 4 4 - 4.5 4.5 - 5 5 - 5.5 5.5 - 6
662 Central Grain Width (mm)
662 Lateral Grain Width (mm)
666 Central Grain Width (mm)
666 Lateral Grain Width (mm)
662 Plant Grain Width (mm)
666 Plant Grain Width (mm)

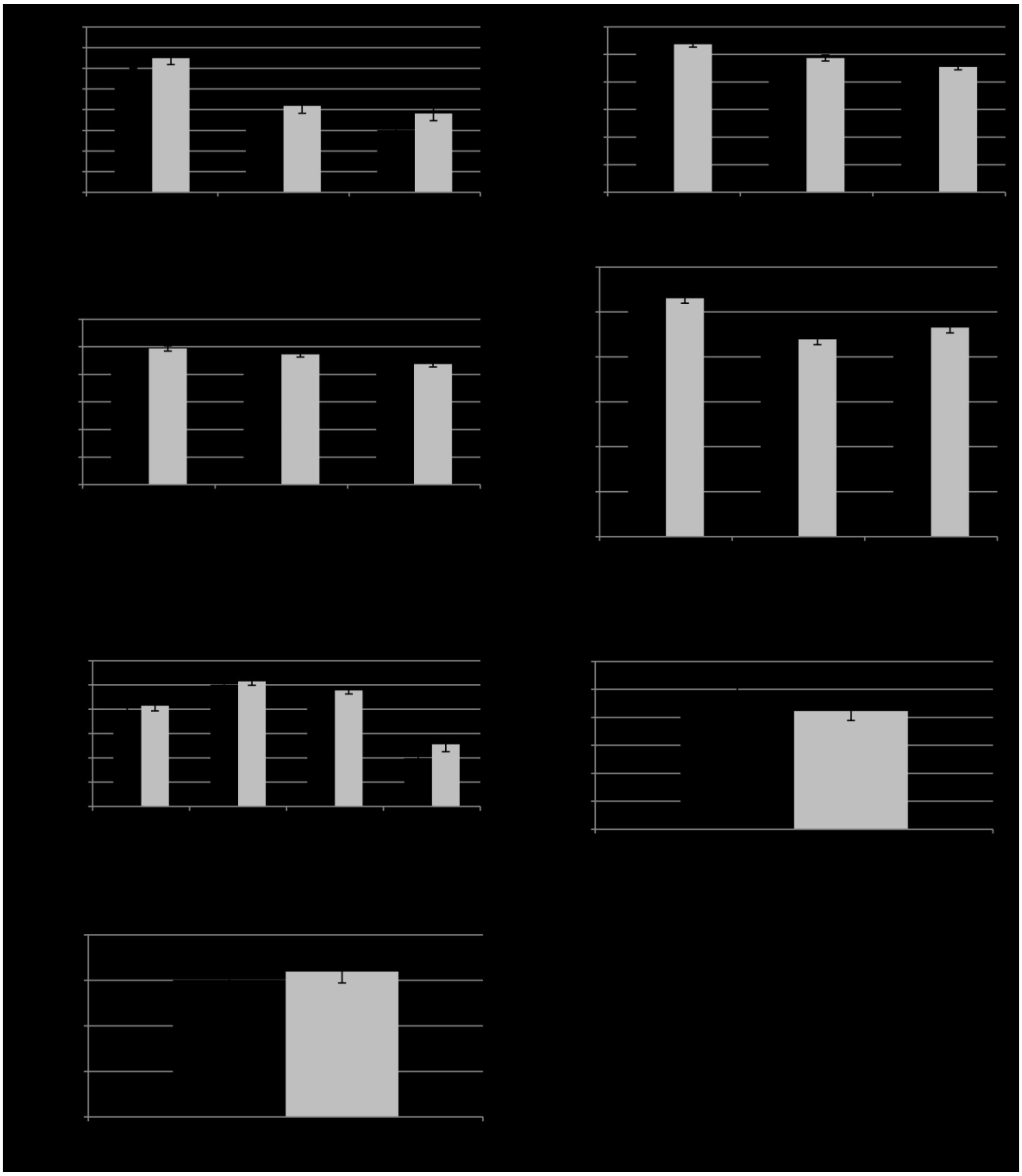

Supplementary Figure 6:

(a) Genotype and phenotype combinations observed within the segregating F2 populations (BW419(*int-a*.1)*Morex and BW902(*vrs3*.f)*Morex)**. (b-q)** Extended analysis of double (662)(*vrs1*.a,*Int-c*.a,*vrs3*) (black bars) and treble homozygous (666)(*vrs1*.a,*Int-c*.a,*vrs3*) (grey bars) six-rowed allele combinations from **(b-e)** glasshouse grown F2 plants and **(f-r)** field grown F3 plots. Graphs **(e-p)** plot data from central and lateral spikelet from the main spikes of five plants and analysis of grain from a sample of 5 whole plants. Error bars are ± S.E.D. TGW, Thousand grain weight.

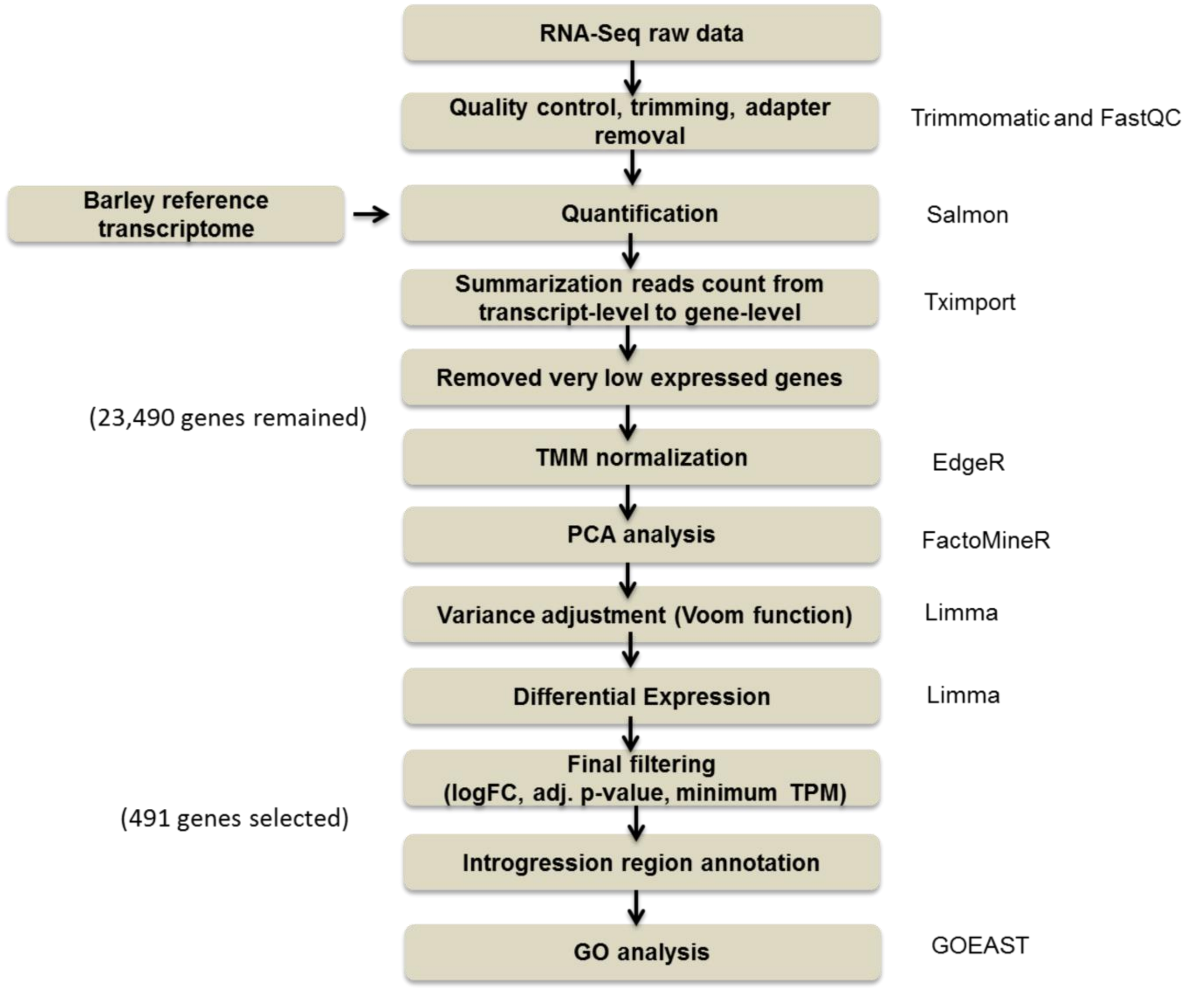

Supplementary Figure 7:

Flow diagram with the main steps (centre) and tools (right side) employed to perform the RNA-Seq analysis.

Supplementary Table 1: Genes within fine-mapping interval

Barley Gene Identifier	Annotation	Orthologous rice locus (based on chromosomal position)
HORVU1Hr1G050780	Tetratricopeptide repeat (TPR)-like superfamily protein	LOC_Os10g42610
HORVU1Hr1G050790	undescribed protein	
HORVU1Hr1G050800	undescribed protein	
HORVU1Hr1G050810	Thymic stromal cotransporter	
HORVU1Hr1G050820	undescribed protein	
HORVU1Hr1G050830	dihydroflavonol 4-reductase-like1	LOC_Os10g42620
HORVU1Hr1G050840	WEB family protein At4g27595	LOC_Os10g42640
HORVU1Hr1G050850	VQ motif-containing protein	LOC_Os10g42650
HORVU1Hr1G050860	Retrotransposon protein	
HORVU1Hr1G050870	undescribed protein	
HORVU1Hr1G050880	Vacuolar protein sorting-associated protein 27	LOC_Os10g42724
HORVU1Hr1G050890	undescribed protein	
HORVU1Hr1G050900	Glycerol-3-phosphate acyltransferase	LOC_Os10g42720
HORVU1Hr1G050910	undescribed protein	
HORVU1Hr1G050920	Phenylalanine--tRNA ligase beta subunit	
HORVU1Hr1G050930	undescribed protein	
HORVU1Hr1G050940	undescribed protein	
HORVU1Hr1G050950	Pentatricopeptide repeat-containing protein	
HORVU1Hr1G050970	shikimate kinase like 2	LOC_Os10g42700
HORVU1Hr1G050960	Inactive poly [ADP-ribose] polymerase RCD1	LOC_Os10g42710
HORVU1Hr1G050980	undescribed protein	
HORVU1Hr1G050990	undescribed protein	
HORVU1Hr1G051000	undescribed protein	
HORVU1Hr1G051010	undescribed protein	LOC_Os10g42690
HORVU1Hr1G051020	xyloglucan endotransglucosylase/hydrolase 28	
HORVU1Hr1G051030	undescribed protein	
HORVU1Hr1G051040	xyloglucan endotransglucosylase/hydrolase 28	LOC_Os10g42670
HORVU1Hr1G051050	Chalcone-flavanone isomerase family protein	LOC_Os10g42660
HORVU1Hr1G051060	cellulose synthase-like D3	LOC_Os10g42750
HORVU1Hr1G051070	Pentatricopeptide repeat-containing protein	LOC_Os10g42760
HORVU1Hr1G051080	26S proteasome non-ATPase regulatory subunit 13 homolog B	
HORVU1Hr1G051090	undescribed protein	
HORVU1Hr1G051100	receptor kinase 3	
HORVU1Hr1G051110	unknown function	
HORVU1Hr1G051120	26S proteasome non-ATPase regulatory subunit 13 homolog B	
HORVU1Hr1G051130	unknown function	
HORVU1Hr1G051140	unknown function	LOC_Os10g42770
HORVU1Hr1G051150	Antiholin-like protein LrgB	LOC_Os10g42780
HORVU1Hr1G051160	Antiholin-like protein LrgB	LOC_Os10g42780
HORVU1Hr1G051180	S-adenosyl-L-methionine-dependent methyltransferases superfamily protein	
HORVU1Hr1G051170	unknown function	
HORVU1Hr1G051190	4-coumarate--CoA ligase-like 3	LOC_Os10g42800
HORVU1Hr1G051200	ERD (early-responsive to dehydration stress) family protein	LOC_Os10g42820
HORVU1Hr1G051210	receptor kinase 2	
HORVU1Hr1G051220	receptor-like kinase 1	
HORVU1Hr1G051230	Orf3 protein	
HORVU1Hr1G051240	Zinc finger BED domain-containing protein RICESLEEPER 1	
HORVU1Hr1G051250	Ribosomal protein L35	LOC_Os10g42860
HORVU1Hr1G051260	WRKY DNA-binding protein 35	LOC_Os10g42850
HORVU1Hr1G051270	undescribed protein	
HORVU1Hr1G051280	NADH:ubiquinone oxidoreductase	LOC_Os10g42840
HORVU1Hr1G051290	sugar transporter 11	LOC_Os10g42830

Supplementary Table 2: *vrs3* mutant allelic series accessions

Allele	Genebank	ID
int-a.1	NordGen	NGB115419
int-a.10	NordGen	NGB115428
int-a.2	NordGen	NGB115420
int-a.64	NordGen	NGB115482
int-a.71	NordGen	NGB115489
int-a.74	NordGen	NGB115492
int-a.77	NordGen	NGB115495
int-a.79	NordGen	NGB115497
int-a.8	NordGen	NGB115426
int-a.86	NordGen	NGB115504
int-a.88	NordGen	NGB115506
int-a.9	NordGen	NGB115427
int-a.14	NordGen	NGB115432
int-a.17	NordGen	NGB115435
int-a.21	NordGen	NGB115439
int-a.27	NordGen	NGB115445
int-a.30	NordGen	NGB115448
int-a.31	NordGen	NGB115449
int-a.32	NordGen	NGB115450
int-a.34	NordGen	NGB115452
int-a.35	NordGen	NGB115453
int-a.37	NordGen	NGB115455
vrs3.f	USDA-ARS National Small Grains Collection	GSHO 774
int-a.102	NordGen	NGB115520
int-a.103	NordGen	NGB115521
int-a.46	NordGen	NGB115464
int-a.51	NordGen	NGB115469
int-a.52	NordGen	NGB115470
int-a.54	NordGen	NGB115472

Supplementary Table 3: Primers spanning *VRS3*

Primer Name	Sequence 5'→3'	Position (bp from predicted ATG start codon)
1F	ATCACCCTCCCCACCTCCCCC	-354
1R	CAATTGGCGTGGTTCGCGGC	224
2F	TTTCTCCGTCCACTTCCGCCG	71
2R	ATGCAACCCCACACGGCAAGG	630
3F	CACCAGCTGCGCTTCTACCCC	505
3R	GCTTCCCTGCCACGACCATCC	1036
4F	GCAGCGAGGGTTTCTACGAATTGC	919
4R	CCCCACAGTTTGCTGGAGTCCG	1437
5F	TTTCGGTTCCACCTATGACC	1136
5R	AATACACTGGCAAGAAAAATGG	1702
6F	AAACTGTGGGGTCAGATTGC	1448
6R	ATTTCACGCCAGAATTCCTC	2097
7F	TGGACGGTGCGCTACTCT	1920
7R	TGATGCTGGTTATGTGATGAA	2599
8F	TTCTTAATTTGGATTTGTCTTGATTG	2399
8R	CTCCACAATTGAAGCCTGTG	2973
9F	GTTTGCATGGCATGTTGAAG	2491
9R	TGGATGTTCAGAATTGATTGTTTT	3142
10F	TTCTGCTTGGTAATTTCTGCAT	2914
10R	TTCAGGGGAAGGCTAGAGAA	3394
11F	TGATCATGGTTATTCCTCATTCC	2550
11R	TTCGCATCAACCTCACAAAG	3192
12F	AAAACAATCAATTCTGAACATCCA	3142
12R	AAGATGTCAACGATGAAGTTGC	3719
13F	TGAAGCAAACTTGCTCGAAG	3667
13R	TCCATGGATTCTCAGTTGGTT	4346
14F	GTGAACTGCTGCTTCCTTGC	4248
14R	TCGCCTTCGTGTAGTTAATGC	4927
15F	ATCTACCGGCAGCTGTTCC	4669
15R	AGACACCATCCCGTCTCAAC	5344
16F	CTCGTAGCGCTGCAAACAG	4761
16R	CCTCATCTGTAACGCATGAAT	5440

Supplementary Table 4: qRT-PCR primers

Primer Name	Sequence 5'→3'	UPL probe number
VRS1_qRT_1F	CCCATAAAATAGCCGAGATAGC	Probe 70
VRS1_qRT_1R	AGGTTTCTGCCGATCTTGAA	
VRS2_qRT_1F	CAACATCGTCGTGTCATCG	Probe 9
VRS2_qRT_1R	GGGAACGAGCCGTAGAGC	
VRS3_qRT_1F	CACTTTCTTTATGAGTGGACGAAA	Probe 101
VRS3_qRT_1R	CAGAAGAGATTTCACGCCAGA	
VRS4_qRT_1F	GTGAACGCCATTAGCACCAT	Probe 77
VRS4_qRT_1R	GTGATCCATCCCAATGCTCT	
INT-C_qRT_1F	ACCATTCCTCCCCTCCATT	Probe 31
INT-C_qRT_1R	GCACCGGCACCGGCACAGAGGTAG	
ACT_qRT_1F	GCGAGTTGTCTGGGTCTTCT	Probe 129
ACT_qRT_1R	ACATGGCAAGGACTTGAGAAA	
HvHOX2_qRT_2F	GGCATCAACAGCATGCATAA	Probe 33
HvHOX2_qRT_2R	CACACGCACGCCTACAGAT	
T6P_qRT_1F	CAAGGTGCTGCGGAAGAG	Probe 105
T6P_qRT_1R	GGTCTCCTTGGCGGACTT	
CKX_qRT_1F	CAGACTTCGCGAGCTTCAC	Probe 79
CKX_qRT_1R	CCCCTCGATGTAGTCGAATG	
TFL1_qRT_1F	TGGTCGAATTTGGTTCAGC	Probe 118
TFL1_qRT_1R	GGCCTTCTGCTAGCTATCAATC	
CKP_qRT_1F	TGGAGGAGGGCTTCATCA	Probe 70
CKP_qRT_1R	TCGAGTTTCTCCATGAGCTG	
TCP_qRT_2F	GCTTGTGCTTGTGAGTGGAA	Probe 35
TCP_qRT_2R	TGCTGGATGGATGATGTGTT	
PDF2_qRT_1F	TGGGTGCAGAAATAACATGC	Probe 7
PDF2_qRT_1R	ATGTTCGGCACTCTGTCCTT	

Supplementary Table 5: Primers for *in situ* hybridisations with T7 promoter sequence in bold.

**Primer name**	**Sequence 5'→3'**
HvVRS3-Forward	TGCTGACTGACTTGGGTTTAG
HvVRS3-Reverse	GCTCCACTTGTTCTCGATGATA
T7-HvVRS3- Forward	**TAATACGACTCACTATAGGGAGA**TGCTGACTGACTTGGGTTTAG
T7-HvVRS3-Reverse	**TAATACGACTCACTATAGGGAGA**GCTCCACTTGTTCTCGATGATA

Supplementary Table 6: Identified mutations across the *vrs3* mutant allelic series (nucleotide position is relative to the ATG start codon)

Allele	Genetic background	Mutation	Mutation type	Reference amino acid
int-a.27	Vrs3.w	G1769A	missense-SNP	G131D
int-a.30	Vrs3.w	G1769T	missense-SNP	G131V
int-a.54	Vrs3.w	G1769A	missense-SNP	G131D
int-a.55	Vrs3.w	G1769A	missense-SNP	G131D
int-a.71	Vrs3.x	G1769A	missense-SNP	G131D
BW902	Vrs3.w	T1774-	Frameshift	C133-
vrs3.f	Vrs3.w	T1774-	Frameshift	C133-
int-a.64	Vrs3.x	CT1856AA	missense-SNP	T160K
int-a.88	Vrs3.x	G1926A	Splice site mutation	
int-a.46	Vrs3.w	G2139T	missense-SNP	V229F
int-a.79	Vrs3.x	G2448A	missense-SNP	G275E
int-a.31	Vrs3.w	C2465T	missense-SNP	L281F
int-a.32	Vrs3.w	C2465T	missense-SNP	L281F
int-a.61	Vrs3.x	C2465T	missense-SNP	L281F
int-a.51	Vrs3.w	T2468A	missense-SNP	Y282N
int-a.2	Vrs3.x	G2474C	missense-SNP	G284R
int-a.34	Vrs3.w	C2524A	nonsense-SNP	Y300STOP
int-a.35	Vrs3.w	C2524A	nonsense-SNP	Y300STOP
int-a.9	Vrs3.w	A2525T	missense-SNP	S301W
int-a.14	Vrs3.w	A2525T	missense-SNP	S301W
int-a.52	Vrs3.w	A2629G	missense-SNP	H306R
int-a.77	Vrs3.x	G2679T	nonsense-SNP	G323STOP
int-a.74	Vrs3.x	A2688T	nonsense-SNP	K326STOP
int-a.17	Vrs3.w	A2823T	nonsense-SNP	K371STOP
int-a.59	Vrs3.x	G2893A	Splice site mutation	
int-a.10	Vrs3.x	A2993G	missense-SNP	E399G
int-a.102	Vrs3.w	G2995A	missense-SNP	A400T
int-a.103	Vrs3.w	G2995A	missense-SNP	A400T
int-a.21	Vrs3.w	G3021A	nonsense-SNP	W408STOP
int-a.8	Vrs3.x	AT3288--	Frameshift	LS497--
BW419	Vrs3.x	CG3464--	Frameshift	R529-
int-a.1	Vrs3.x	CG3464--	Frameshift	R529-
int-a.86	Vrs3.x	G3471A	missense-SNP	C531Y

Supplementary Note

Plant Ontogeny

Phenotypic evaluation of the spikes from four F2 populations, Bowman*BW419(*int*-a.*1*), Bowman*BW902(*vrs3*.f), Barke*BW419(*int*-a.*1*) and Barke*BW902(*vrs3*.f), showed that the homozygous *vrs3* individuals from the BW902 populations exhibited greater levels of awn development on the lateral spikelets from the two basal spikelet node positions compared to the *vrs3* homozygous individuals from the BW419(*int*-a.*1*) population (Basal spikelet node 1: χ^2= 16.87, 5 d.f. P = 0.005, Basal spikelet node 2: χ^2= 12.47,5 d.f. P = 0.025). Across all four positions sampled along the spike, the homozygous *vrs3* spikes from the Bowman populations showed a greater association with lateral awn development than those from the Barke populations, illustrating the genotype dependence of the phenotypic penetrance.

Analysis of lateral spikelet grain fill showed a similar trend to lateral awn development. The Bowman*BW419 and Barke*BW419 populations showed no significant association between the basal spikelet node internodes and lateral spikelet grain fill, suggesting that at this position, the BW419(*int*-a.*1*) homozygous *vrs3* F2 spikes resemble a two-rowed phenotype (BW419Ba (χ^2=2.5, 1d.f., P = 0.293) and BW419Bo (χ^2=6.45, 1d.f., P = 0.057)). However, the BW902 populations showed a significant association between the basal spikelet node and lateral spikelet grain fill, indicating that BW902(*vrs3*.f) exhibits a stronger mutant phenotype with lateral spikelet grain fill persisting further down the spike. Across the extended mutant series, lateral spikelet grain fill varied considerably amongst individuals (max. 56.5%, min. 0.6%). Variation in lateral grain fill was also observed between mutant alleles at the same nucleotide position. Mutations that were generated and remain present in different genetic backgrounds show different phenotypic penetrance, i.e. *int-a*.27 (Foma): 6.8%, *int-a*.71(Bonus): 8.7%, *int-a*.54 (Kristina): 38.5%.

Across all mutants, no significant difference in days to ear emergence (flowering) between mutant and wild type was identified. However, multivariate analysis suggested that lateral spikelet grain fill was enhanced in *vrs3* mutant lines whose genetic background resulted in earlier ear emergence.

Phylogeny

Comparative sequence analysis identified orthologues of *VRS3* across grass species. The rice *VRS3* partial orthologue Os*JMJ706* (LOC_Os10g42690) was previously functionally characterised as an H3K9me2/me3 demethylase[1]. Both sequence homology and conservation of synteny therefore suggest that this is also the function of *VRS3*. Reciprocal BLAST of *VRS3* identified a paralogue within Barley (HORVU6Hr1G067480 = MLOC_53868.2), which was also found in other grass species, and the chromosomal locations of the paralogues are consistent with a duplication event in a common ancestor to the Poaceae rather than separate intraspecific duplications. Although sharing a syntenic chromosomal location with *VRS3*, phylogenetically *JMJ706* is atypically positioned ancestrally, possibly indicative of directional selection at this locus. Genome wide association studies have

associated *JMJ706* with increased primary and secondary branching in the rice inflorescence[2] . Panicle branching represents a key yield-related trait in rice that may explain the increased sequence diversity at this locus. The phylogeny presented here is consistent with that of a detailed phylogenetic study of JmjC domain containing proteins across plant species, which placed Arabidopsis (AT5G46910), rice and maize loci within the plant-specific *PKDM8*, H3K9 demethylase clade[3] and *REF6* and *ELF6* within the sister *PKDM9*, H3K27me3 clade. The H3K9 methylation marks H3K9me2 and H3K9me3 are repressive marks associated with gene silencing.

Gene expression analysis

Additional methods

Barley Atlas gene expression

An RNA-seq based atlas of gene expression from 16 tissues from the six-row cultivar Morex[4,5] is available at http://camel.hutton.ac.uk/barleyGenes_JLOC2/. The atlas provides transcript abundance data (FPKM values) of predicted genes from the whole-genome-shotgun sequence from the six-row barley cv. Morex. FPKM values for *VRS1-VRS5* genes across the tissue series are shown in **Supplementary Fig. 3**. *(For review, login: JHI-barleyGenes, Password: JHI-barleyGenes)*

RNA-seq bioinformatic analysis. A total of 914,782,574 pairs of reads with the read length of 150bp were generated from 32 samples. For quality control and removal of adapters we used FASTQC (version 0.11.3) and Trimmomatic (version 0.30)[6]. The minimum phred quality score was 20 and the minimum length of the reads accepted after trimming was 75 base pairs. Reads that remained unpaired after QC were excluded from further analysis. Nearly 95% of the reads remained for downstream analysis.

Quantification of the transcripts was carried out using Salmon version 0.7.2[7]. Transcripts from both high and low confidence genes (see 5) were used as the transcript reference, and k-mer of 31 and quasi-mapping mode were chosen for building the index in Salmon. In the Salmon quantification step, --seqBias –gcBias were specified to adjust for sequence specific and GC content biases.

The transcript quantification output from Salmon was imported and summarized from transcript-level to gene-level read counts using the R package *tximport* version 1.2.0[8]. countsFromAbundance was set as default. The summarized gene read counts were then converted to count per million reads (CPM). Genes with CPM>1 in at least 6 samples were considered as expressed and were selected for further analysis. The data were then normalized using weighted trimmed Mean of M values (TMM) method to account for the raw library sizes using function *calcNormFactors* (calculate normalization factors to scale the raw library sizes) in EdgeR version 3.16.5[9]. Principle Component Analysis (PCA)[10] was carried out to examine batch effects and no technical issues were identified. The normalized CPM values were then transformed into log2-reads-per-million (log2CPM), which were used to estimate the relationships between mean and variance and generate weights for variance adjustments using the *voom* function in Limma package version 3.30.9[11,12]. After that, a general linear model was set up using genotype and development stages (Bowman WT and BW902(*vrs3*.f), awn primordium (AP, spike length 5mm) and white anther (WA, spike length 10mm) stages as factors and contrast groups BW902.AP vs WT.AP and BW902.WA vs WT.WA were set to

examine the differentially expressed genes between specific subgroups. The p values were adjusted for multiple testing using Benjamini & Hochberg (BH)[13] method. The final lists of differentially expressed genes for each contrast were produced using the following criteria: 1) adjusted p <0.05) $|\log_2$ fold change$| \geq 0.5$ and 3) the expression values of that gene ≥ 2TPM in at least 6 samples. All P values quoted in the manuscript to interpret and analyse the data are adjusted P values by BH method to control false discovery rate. The complete list of differentially expressed genes between Bowman and BW902(*vrs3*.f), including those that lay within the introgression in BW902(*vrs3*.f) (491 genes), is shown in **Supplementary Data 1b and 1c**. The level of expression of these genes across the 2 comparisons is shown in **Supplementary Data 1a**. Genes present in the introgression regions are identified and annotated in **Supplementary Data 1b and 1c**. Biological interpretation of the differentially expressed genes focused on those outside of the introgression (364 genes) to focus on the downstream effects of the mutation.

Gene Ontology enrichment analysis. 187 of the 491 differentially expressed genes have GO terms associated (**Supplementary Data 1d**). Gene ontology enrichment analysis was performed with GOEAST[14]. Customized analyses were run in default (hypergeometric test, 0.1 for false discovery rate, minimum 5 of associated genes in reference (m value) for display). The customized gene annotation dataset was the list of 23,636 annotated genes with GO codes associated. Some GO terms were found overrepresented (**Supplementary Data 1e**).

*Quantitative PCR validation***.** RNA-seq results for key genes were independently validated by qRTPCR. Plants of Bowman WT and BW902(*vrs3*.f) were grown in the glasshouse in a randomised design, under the same conditions as for the RNA-seq experiment. Three biological repetitions per genotype with 10 spikes for the AP (5mm spikes) stage and 8 spikes for the WA (10mm spikes) stage each were harvested. RNA was isolated as described in the Online Methods. Reverse transcription and cDNA synthesis were performed using SuperScript III Reverse Transcriptase (Invitrogen). Quantitative PCR was carried out Universal Probe Library (Roche) hydrolysis probes on the Applied Biosystems StepOnePlus machine. qRT-PCR primer sequences are listed in **Supplementary Table 4**.Three technical repeats were performed for each cDNA-primer combination per sample. qRT-PCR data were obtained using SDS2.3 software (Applied Biosystems) and analysed by $2^{-\Delta CT}$ method. Significant differences among the samples were calculated using Student's t-test (two-tailed). The qRT-PCR validation of differentially expressed genes identified in the RNA-seq experiment are given in **Supplementary Fig. 5.**

Biological analysis.

While some of the differentially expressed (DE) genes between Bowman and BW902(*vrs3*.f) are known to control inflorescence morphology, we detected additional DE genes which may be relevant to the *vrs3* phenotype. Apart from *VRS1*, other HD-Zip class I genes (*HvHOX*) proteins that have diverse functions in regulating adaptive plant growth in conjunction with phytohormones[15,16,17], were significantly downregulated in AP (5mm-spike) inflorescences, including orthologues of rice *OsHOX16* (HORVU6Hr1G072810, LFC= -0.549, adjusted adjusted P= 0.014) and *OsHOX21* (HORVU4Hr1G075180, LFC= -0.569, adjusted P= 0.008). The paralogue of *VRS1*, *HvHOX2* was downregulated in WA (10mm-spike) inflorescence (HORVU2Hr1G036680, LFC= -0.946; adjusted P= 0.049) and HORVU6Hr1G075650, a *PROLIFEREATING CELL FACTORs (PCF)* class I TCP[18] was

downregulated at both stages (LFC= -0.719, adjusted P= 0.015 (AP); LFC= -0.596, adjusted P= 0.006 (WA)) (**Supplementary Fig. 5d**).

Molecular control of hormone metabolism was perturbed between wild type and mutant. In addition to predicted changes in jasmonate, cytokinin metabolism and signalling in BW902(*vrs3*.f) , we found *APETALA2-like/Ethylene Responsive Factor* (*AP2-like/ERF*) genes downregulated. Genes that belong to *AP2-like/ERF* family have been reported to play an important role in the spikelet meristem determinacy and floral organ identity in cereals[19]. In our data, we detected HORVU7Hr1G037180, an *ERF* transcription factor that was significantly downregulated in both stages (LFC= -0.694 adjusted P= 0.004 (AP); LFC= -0.568adjusted P= 0.006 (WA)). The function of the putative ortholog of this gene in rice (*OsAP2/EREBP-132*) (*DREB2B*) seems to be related to drought resistance and heat-shock stress tolerance[20,21]. We also found HORVU5Hr1G045290, another *AP2-like/ERF*, with significant DE in WA spikes (LFC= -0.614; adjusted P= 0.023) possibly suggesting that it acts further downstream in the developmental pathway. *Arabidopsis* and rice orthologs of this gene belong to the *WRINKLED 1* family, a key regulator of seed oil biosynthesis and seed storage metabolism in *Arabidopsis* and maize where it is involved in glycolysis and fatty acid biosynthesis in the plastids[22,23]. In maize, expression of one *WRI1* orthologs (*ZmWRI1a*) has been detected in immature and mature tassels[23].

Sugar metabolism also appears influenced by *VRS3*. While we observed a significant reduction in the expression of *T6PP* in AP BW902(*vrs3*.f) inflorescences, we found three sugar transporter *SWEET* genes were significantly DE in BW902(*vrs3*.f) spikes at WA stage: HORVU5Hr1G076770 and HORVU7Hr1G054710 were significantly downregulated (LFC= -1.106, adjusted P= 5.03 10^{-4} and LFC= -1.147, adjusted P= 0.004, respectively) while HORVU3Hr1G107780 (LFC= 0.736; adjusted P= 0.002) was significantly upregulated. In the barley atlas dataset all three are highly expressed in developing inflorescence/caryopsis tissues. *SWEET* genes (also known as *saliva* or *MtN3*) encode transmembrane proteins involved in sugar transport. In plants, they participate in a wide range of the biological processes, such as host-pathogen interactions, reproductive development, senescence, and abiotic stress responses[24]. HORVU5Hr1G076770 and HORVU7Hr1G054710 are orthologs of *Xa13/Os8N3/OsSWEET11* in rice, where supressed expression causes reduced fertility or sterility due to compromised microspore development, resulting in the gradual degeneration of immature pollen[24,25]. Here, downregulation was significant in WA BW902(*vrs3*.f) spikes, the opposite of what may be expected based on the data from rice. However, the *SWEET* gene HORVU3Hr1G107780 was significantly upregulated at the same stage, consistent with the effect of *SWEET* genes in rice inflorescence[26]. The contrasting regulation we observe here suggests that different *SWEET* genes may play different roles in determining barley spike architecture and pollen fertility.

Stress metabolism and signalling genes were also DE. We observed thionin-encoding genes (also known as *PR13* family) to be significantly downregulated exclusively in WA BW902(*vrs3*.f) spikes. These genes are known to be involved in plant defence. Differences in the expression of thionins throughout inflorescence development has been described previously[27,28] and downregulation in WA BW902(*vrs3*.f) spikes may indicate a specific role in certain stages of barley spike development. *Glutathione S-transferase* (*GST* family), a group of genes related to stress signalling were also significantly downregulated in BW902(*vrs3*.f). HORVU4Hr1G081170 (LFC= -1.225, adjusted P= 0.003 (AP); LFC= -0.927, adjusted P= 4.68 10^{-4} (WA)) and HORVU4Hr1G081150 (LFC= -0.7811 adjusted P=

0.04 (AP); LFC= -0.801, adjusted P= 0.004 (WA)) putative orthologs of *OsGSTF14* and *OsGSTF15* in rice respectively, are strongly expressed in the panicle[29]. The lower expression found in the *vrs3* inflorescence may suggest that these genes could act as a regulators of spike development and meristem determinacy in barley.

We detected DE of genes involved in meristem determinacy such as *MADS-box* TFs in BW902(*vrs3*.f). HORVU3Hr1G026650, an *AGAMOUS-like protein-1* (*AG1*) ortholog of *OsMADS3* in rice[30] was significantly upregulated in both developmental stages of BW902(*vrs3*.f) spikes studied (LFC= 0.7005, adjusted P= 1.61 10^{-9} (AP); LFC= 0.653, adjusted P= 7.73 10^{-9} (WA)) whereas HORVU3Hr1G095240, an ortholog of *OsMADS65* in rice[30] (LFC= 0.983, adjusted P= 0.002) was only significantly upregulated in the AP stage. In rice, *OsMADS65* shows high expression in vegetative and in reproductive tissues, but *OsMADS3* rises with the development of the inflorescence and continues increasing during seed development[30], potentially indicating a role in determining meristem identity. Finally, a homolog of *TERMINAL FLOWER 1-LIKE* (*TFL1-LIKE*) (HORVU4Hr1G078770), previously implicated in modifying inflorescence architecture in cereals[31,32] was significantly downregulated at AP and WA stages (LFC= -3.292 adjusted P= 5.42 10^{-4} (AP); LFC= -3.237, adjusted P= 6.10 10^{-7} (WA)) in the mutants spikes, suggesting additional roles of this gene family in spike morphology.

Field /glasshouse trial

Genotyping of F2 individuals used allele specific KASP assay designed to diagnostic polymorphisms within the *Vrs1*, *VRS5*(*Int-c*) and *Vrs3* genotypes. 27 different genotype combinations segregated as would be expected for an F2 population with three segregating genes. These combinations could be divided into five different phenotypic classifications (**Supplementary Fig. 6**).

Field-grown plants

Each plot comprised a paired row of a 20 plant F3 family derived from a single F2 plant homozygous for the *VRS1*, *VRS5*(*INT-C*) and *VRS3*. At Zadoks growth stage 89 (hard-grain), a sample of five plants was harvested per plot. The main spike of each plant was separated into central and lateral grain. The remaining spikes on the five plants were threshed as a bulk to determine grain size variation across all tiller types.

We observed significant increases in grain width in the 666 genotype compared to the 662 genotype across the three grain fractions, central, lateral and whole plant (**Supplementary Fig. 6**). In contrast, to the glasshouse environment, we found only a significant increase in lateral grain area. The lateral:central grain area ratio marginally increased under field conditions in the 666 allele combination compared to the 662 combination, 0.91 compared to 0.90, but in this environment the difference was not significant. Average grain size was smaller in the glasshouse environment than the field indicative of more limiting conditions. This may suggest that the incorporation of *vrs3* within the spring six-rowed barley model would be most beneficial in environments where grain fill is impaired such as high temperatures or drought post-anthesis.

Supplementary References

[1]Sun, Q., & Zhou, D.X. Rice jmjC domain-containing gene *JMJ706* encodes H3K9 demethylase required for floral organ development. *Proc. Natl. Acad. Sci. USA* **105,** 13679–13684 (2008).

[2]Rebolledo, M.C. *et al.* Combining image analysis, genome wide association studies and different field trials to reveal stable genetic regions related to panicle architecture and the number of spikelets per panicle in rice. *Front. Plant Sci*. **7,** 1384 doi.org/10.3389/fpls.2016.01384 (2016).

[3]Qian, S., Wang Y., Ma H., Zhang L. Expansion and functional divergence of Jumonji C-containing histone demethylases: significance of duplications in ancestral angiosperms and vertebrates. *Plant Physiol*. **168**, 1321-1337 (2015).

[4]The International Barley Genome Sequencing Consortium. A physical, genetic and functional sequence assembly of the barley genome. *Nature* **491**, 711–716 (2012).

[5]Mascher, M. *et al.* A chromosome conformation capture ordered sequence of the barley genome. *Nature* **544**, 427-433 (2017). (2017).

[6]Bolger, A.M., Lohse, M., Usadel, B. Trimmomatic: a flexible trimmer for Illumina sequence data. *Bioinformatics* **30**, 2114-2120 (2014).

[7]Patro, R. *et al*. Salmon provides accurate, fast, and bias-aware transcript expression estimates using dual-phase inference. Preprint at *bioRxiv* https://doi.org/10.1101/021592 (2016).

[8]Soneson, C., Love, M.I. & Robinson, M.D. Differential analyses for RNA-seq: transcript-level estimates improve gene-level inferences. *F1000Research* **4** (2015).

[9]Robinson, M.D., McCarthy, D.J. & Smyth, G.K. edgeR: a Bioconductor package for differential expression analysis of digital gene expression data. *Bioinformatics* **26**, 39-40 (2010).

[10]Le, S., Josse,J., & Husson, F. FactoMineR: An R Package for Multivariate Analysis. *J. Stat. Softw.* **25**, 1-18 (2008).

[11]Law, CW et al. Voom: precision weights unlock linear model analysis tools for RNA-seq read counts. *Genome Biol.* **15**, R29 doi: 10.1186/gb-2014-15-2-r29 (2014)

[12]Ritchie ME *et al.* Limma powers differential expression analyses for RNA-sequencing and microarray studies. *Nucleic Acids Res.* **43**, e47 (2015).

[13]Benjamini, Y. & Hochberg, Y. Controlling the false discovery rate: a practical and powerful approach to multiple testing. *J. R. Stat. Soc. Series B* **57**, 289–300 (1995).

[14]Zheng, Q., & Wang, X.J. GOEAST: a web-based software toolkit for Gene Ontology enrichment analysis. *Nucleic Acids Res.* **36**, W358-W363 (2008).

[15]Agalou, A. *et al*. A genome-wide survey of HD-Zip genes in rice and analysis of drought-responsive family members. *Plant Mol. Biol.* **66**, 87–103 (2008).

[16]Jain, M., Tyagi, A.K., & Khurana, J.P. Genome-wide identification, classification, evolutionary expansion and expression analyses of homeobox genes in rice. *FEBS J.* **275**, 2845-2861 (2008).

[17]Zhao Y. *et al*. Systematic analysis of sequences and expression patterns of drought-responsive members of the HD-Zip gene family in maize. *PloS one* **6**, e28488 doi.org/10.1371/journal.pone.0028488 (2011).

[18]Martín-Trillo, M. & Cubas, P. TCP genes: a family snapshot ten years later. *Trends Plant Sci*. **15**, 31-39 (2010).

[19]Thompson, B.E. & Hake S. Translational biology: from *Arabidopsis* flowers to grass inflorescence architecture. *Plant Physiol.* **149**, 38-45 (2009).

[20]Rashid, M., Guangyuan, H., Guangxiao, Y., Hussain, J. & Yan X. AP2/ERF transcription factor in rice: genome-wide canvas and syntenic relationships between monocots and eudicots. *Evol. Bioinform. Online* **8**, 321–355 (2012).

[21]Matsukura, S. *et al.* Comprehensive analysis of rice *DREB2*-type genes that encode transcription factors involveed in the expression of abiotic stress responsive genes. *Mol. Genet. Genomics* **283**, 185-196 (2012).

[22]Cernac, A. & Benning, C. *WRINKLED1* encodes an AP2/EREB domain protein involved in the control of storage compound biosynthesis in *Arabidopsis*. *Plant J.* **40**, 575-585 (2004).

[23]Pouvreau, B. *et al.* Duplicate maize *Wrinkled1* transcription factors activate target genes involved in seed oil biosynthesis. *Plant Physiol.* **156**, 674-686 (2011).

[24]Yuan, M. & Wang, S. Rice *MtN3/saliva/SWEET* family gene and their homologues in cellular organisms. *Mol. Plant* **6**, 665–674 (2013).

[25]Chu, Z. *et al.* Promoter mutations of an essential gene for pollen development result in disease resistance in rice. *Genes Dev.* **20**, 1250–1255 (2006).

[26]Yuan, M., Chu, Z., Li, X., Xu, C. & Wang, S. The bacterial pathogen *Xanthomonas oryzae* overcomes rice defenses by regulating host copper redistribution. *Plant Cell* **22**, 3164–3176 (2010).

[27]Urdangarín, M.C., Norero, N.S., Broekaert, W.F., & de la Canal, L. A defensin gene expressed in sunflower inflorescence. *Plant Physiol. Biochem*. **38**, 253-258 (2000).

[28]Tregear, J.W. *et al.* Characterization of a defensin gene expressed in oil palm inflorescences: induction during tissue culture and possible association with epigenetic somaclonal variation events. *J. Exp. Bot.* **53**, 1387-1396 (2002).

[29]Jain, M., Ghanashyam, C. & Bhattacharjee, A. Comprehensive expression analysis suggests overlapping and specific roles of rice *glutathione S-transferase* genes during development and stress responses. *BMC Genomics* **11**, 73 doi: 10.1186/1471-2164-11-73 (2010).

[30]Arora, R. *et al.* MADS-box gene family in rice: genome-wide identification, organization and expression profiling during reproductive development and stress. *BMC Genomics* **8**, 242 doi: 10.1186/1471-2164-8-242 (2007).

[31]Danilevskaya, O.N., Meng, X. & Ananiev, E.V. Concerted modification of flowering time and inflorescence architecture by ectopic expression of *TFL1*-like genes in maize. *Plant Physiol.* **153**, 238-251 (2010).

[32]Nakagawa, M., Shimamoto, K. & Kyozuka, J. Overexpression of *RCN1* and *RCN2*, rice *TERMINAL FLOWER 1/CENTRORADIALIS* homologs, confers delay of phase transition and altered panicle morphology in rice. *Plant J.* **29**, 743-750 (2002).
