# Supplementary material for: Barley SIX-ROWED SPIKE3 encodes a putative Jumonji C-type H3K9me2/me3 demethylase that represses lateral spikelet fertility
Source: Nat Commun. 2017 Oct 16;8:936. doi: 10.1038/s41467-017-00940-7 (PMC5643332; doi:10.1038/s41467-017-00940-7)
Supplement: Supplementary file 3 — Description of Additional Supplementary Files [file 41467_2017_940_MOESM3_ESM.pdf]

**File name**: Supplementary Information
**Description**: Supplementary Figures, Supplementary Tables, Supplementary Note and Supplementary References

**File name**: Supplementary Data 1
**Description**: Differential Gene Expression and GO Enrichment

**File name**: Supplementary Data 2
**Description**: KASP allele specific PCR target polymorphisms and sequences

**File name**: Supplementary Data 3
**Description**: Protein sequences used for Phylogenetic analysis

**File name**: Supplementary Data 4
**Description**: 22 cultivars fully sequenced for VRS3

**File name**: Supplementary Data 5
**Description**: Geolocations and Vrs3 haplotypes of Wild and Landrace Barley accessions

**File name**: Supplementary Data 6
**Description**: *Vrs3* haplotype diversity across the landrace and wild germplasm surveyed

**File name**: Peer Review File
